# ﻿Morphological and molecular characterisation of *Mordellistenapeloponnesensis* Batten, 1980 (Coleoptera, Mordellidae), with first records from Italy and Turkey

**DOI:** 10.3897/zookeys.1214.133348

**Published:** 2024-10-01

**Authors:** Dávid Selnekovič, Ján Kodada, Nilay Gülperçin, Serdar Tezcan, Enrico Ruzzier

**Affiliations:** 1 Department of Zoology, Faculty of Natural Sciences, Comenius University in Bratislava, Ilkovicočova 6, Bratislava, SK-842 15, Slovakia; 2 Natural History Application and Research Centre, Ege University, 35040, Izmir, Turkey; 3 Department of Plant Protection, Faculty of Agriculture, Ege University, 35040, Izmir, Turkey; 4 Department of Science, Università Roma Tre, viale G. Marconi 446, 00146 Rome, Italy; 5 National Biodiversity Future Center (NBFC), 90133 Palermo, Italy

**Keywords:** Distribution, DNA barcoding, haplotype, Mediterranean, morphology, tumbling flower beetles

## Abstract

*Mordellistenapeloponnesensis* Batten, 1980, previously known from Cyprus and Greece, is reported from Italy and Turkey for the first time. The species is redescribed based on type specimens and additional material from its entire known distributional range. Eighteen DNA barcoding sequences of *M.peloponnesensis* from Greece, Cyprus, and Italy were generated, and genetic variability across the sampling localities was examined. Three mitochondrial haplotypes were detected within *M.peloponnesensis*. Specimens from mainland Italy share the same haplotype as those from Rhodes and Cyprus, whereas Sardinian specimens exhibit a distinct haplotype. The third haplotype is represented by one specimen from Cyprus. The DNA barcoding sequences of *M.peloponnesensis* were compared with those of the morphologically allied *M.gemellata* Schilsky, 1898, and *M.pyrenaea* Ermisch, 1966, to reveal the phylogenetic relationships between the species.

## ﻿Introduction

*Mordellistena* A. Costa, 1854, is the largest genus within Mordellidae Latreille, 1802, with more than 150 species known to occur in Europe (Selnekovič and Kodada 2019; Horák 2020; Selnekovič et al. 2021a; Selnekovič et al. 2023; Ruzzier and Di Giulio 2024). Due to their high diversity and the limited number of experts studying this group in the past, current sampling efforts continuously yield new data on the distribution of tumbling flower beetles in Europe (e.g., Ruzzier 2013; Selnekovič and Ruzzier 2019; Ruzzier et al. 2020; Selnekovič et al. 2021b; Selnekovič and Kodada 2022). *Mordellistenapeloponnesensis* Batten, 1980 was initially described based on specimens collected in southern mainland Greece and the Peloponnese (Batten 1980) and later was reported also from Cyprus (Horák 2008, 2020). During collecting trips in Sardinia and mainland Italy between 2021 and 2023, we collected 13 specimens that morphologically correspond to *M.peloponnesensis*. In addition, subsequent examination of specimens from the collections of the National Museum in Prague, the University of Ege in Izmir as well as the E. Ruzzier collection provided additional specimens of *M.peloponnesensis* from Italy (Apulia and Sicily) and Turkey.

Our records significantly expand the known distribution of *M.peloponnesensis*, representing the northernmost and westernmost occurrences of the species. The study of extensive material, including type series, allowed us to provide a redescription of the species and a differential diagnosis to separate it from the morphologically allied species of the *M.gemellata* species group. We also explored the genetic variability of *M.peloponnesensis* across the sampled localities, showing that individuals from the Italian mainland share the same mitochondrial haplotype with individuals from Rhodes and Cyprus, whereas the Sardinian specimens exhibit a distinct, well-separated haplotype. The COI barcoding sequences generated during this study represent the first available DNA sequences for the species.

## ﻿Material and methods

For this study, 108 individuals of *M.peloponnesensis* from Cyprus, Greece, Italy, and Turkey were examined, including 18 paratypes. Material is deposited in the following institutions and private collections: Finnish Museum of Natural History, Helsinki, Finland (**MZH**); Hungarian Natural History Museum, Budapest, Hungary (**HNHM**); Lodos Entomological Museum, Izmir, Turkey (**LEMT**); Museum für Naturkunde der Humboldt-Universität, Berlin, Germany (**MNB**); National Museum, Prague, Czechia (**NMPC**); Naturalis Biodiversity Centre, Leiden, The Netherlands (**NBCL**); Senckenberg Naturhistorische Sammlungen, Dresden, Germany (**SNSD**); collection of Dávid Selnekovič, Bratislava, Slovakia (**DSBS**); collection of Enrico Ruzzier, Mirano, Italy (**ERPC**), and collection of Marion Mantič, Hlučín-Bobrovníky, Czechia (**MMCZ**).

Specimens of *M.peloponnesensis* were compared with the type series of other species that are morphologically associated with the *M.gemellata* group: *M.algeriensis* Ermisch, 1966 (SNSD), *M.aureotomentosa* Ermisch, 1977 (SNSD), *M.elbrusicola* Ermisch, 1969 (SNSD), *M.gemellata* Schilsky, 1899 (MNB), *M.maroccana* Ermisch, 1966 (SNSD), *M.microgemellata* Ermisch, 1965 (SNSD), *M.pyrenaea* Ermisch, 1966 (SNSD), *M.rhenana* Ermisch, 1956 (SNSD), *M.wankai* Ermisch, 1966 (SNSD), and *M.zoltani* Ermisch, 1977 (HNHM).

Individuals used for DNA isolation were killed in 96.3% ethanol and stored at -20 °C. DNA isolation and amplification procedures followed those provided by Selnekovič et al. (2023). The COI gene was amplified by PCR using standard primer pairs LCO1940 and HCO2198 (Folmer et al. 1994) or LEP-F1 and LEP-R1 (Hebert et al. 2003). After DNA isolation, the specimens were soaked for several hours in 5% acetic acid at room temperature, dissected, and mounted on cards. The dissected genitalia were cleared in lactic acid for several days, then dehydrated in 96.3% ethanol and mounted on slides in Euparal (Paradox Co., Cracow, Poland). After examination, the genitalia were mounted on the card with the respective specimen using dimethyl hydantoin formaldehyde (Entomopraxis, Barcelona, Spain).

Specimens were observed under an M205 C stereomicroscope (Leica, Wetzlar, Germany) with magnification up to 120× and diffused LED light (6400 K). Photographs of habitus were taken with an EOS 5D Mark II camera (Canon, Tokyo, Japan) attached to an Axio Zoom.V16 stereoscope (Zeiss, Oberkochen, Germany); photographs of genitalia were taken with an Axio Imager.M2 microscope (Zeiss, Oberkochen, Germany). The images were stacked using Zerene Stacker v.1.4 software (https://zerenesystems.com/cms/stacker) and edited in Adobe Photoshop CC (https://www.adobe.com/products/photoshop.html) and DxO Photolab 5 (https://www.dxo.com/dxo-photolab/). Measurements were made with an ocular micrometre in the M205 C stereomicroscope and are given in the text as the range, followed by the arithmetic mean and standard deviation enclosed in parentheses. The measured characters are abbreviated in the text as follows:

**EL** elytral length from scutellar apex to elytral apices along suture;

**EW** maximum elytral width;

**HL** head length from anterior clypeal margin to occipital carina along midline;

**HW** maximum head width;

**LPrL** maximum left paramere length;

**PL** pronotal length along midline;

**PW** maximum pronotal width;

**PygL** maximum pygidial length;

**RPrL** maximum right paramere length;

**TL** combination of head, pronotal and elytral lengths

Nucleotide sequences were trimmed and assembled into contigs using ChromasPro v.2.1.10 (Technelysium Pty Ltd, South Brisbane, Queensland, Australia). The final alignment was performed manually in Mesquite v.3.81 (Maddison and Maddison 2023), and it is available in the Suppl. material 1. The uncorrected pairwise distances (p-distances) were calculated using the MEGA11 software (Tamura et al. 2021). Maximum likelihood analysis (ML) was performed using IQ-TREE (Nguyen et al. 2015). Three partitions were defined based on the codon positions. The best substitution models (TNe, F81+F, HKY+F) were identified by the built-in ModelFinder according to the BIC criterion. Node support values were obtained from 1000 ultrafast bootstrap replicates and tested with the aBayes test (Anisimova et al. 2011). The resulting trees were subsequently rooted in FigTree v.1.4.4 (https://github.com/rambaut/figtree). *Natirricahumeralis* (Linnaeus, 1758) was selected as the outgroup, as preliminary phylogenetic analyses indicate that the genus *Natirrica* is a sister group to *Mordellistena*. A haplotype network was created using the pegas 1.3 library (Paradis 2010).

All voucher specimens used in this study, along with respective BOLD Sample and Process IDs and GenBank accession numbers, are listed in the linked data table (Suppl. material 2). Trace files and additional information about voucher specimens are available within a dataset on BOLD (dx.doi.org/10.5883/DS-MPELOP).

## ﻿Results

### ﻿ Mordellistena (s. str.) peloponnesensis

Taxon classificationAnimaliaColeopteraMordellidae

Batten, 1980

77607973-5E11-51FB-82AE-2983CEFCD545

Figs 1 , 2

Mordellistena peloponnesensis Batten, 1980: 42–44. Type locality: “Skála, Pelopónnesos, Greece”.
Mordellistena (s. str.) peloponnesensis : Horák (2008, 2020).

#### Type material examined.

***Paratypes.*** Greece • 3 males; Lakonia, Skala env. [same as holotype]; 9 Jul. 1978; R. Batten leg.; NBCL, RMNH.INS.1485999 [genitalia illustrated], RMNH.INS.1486000, RMNH.INS.1486001 • 4 males; Arkadhia, Tripolis env.; 5 Jul. 1977; R. Batten leg.; NBCL, RMNH.INS.1485986, RMNH.INS.1485987, RMNH.INS.1485988, RMNH.INS.1485989 • 1 male; Messinia, 2 km N. of Kardamili; 6 Jul. 1977; R. Batten leg.; NBCL, RMNH.INS.1485985 • 2 males; Messinia, Pilos env.; 8 Jul. 1978; R. Batten leg.; NBCL, RMNH.INS.1485996, RMNH.INS.1485997 • 1 male; Korinthia, Neméa env.; 10 Jul. 1978; R. Batten leg.; NBCL, RMNH.INS.1485990 • 1 male; same data as preceding; HNHM • 1 male; Ilia, 4 km E. of Pirgos; 6 Jul. 1977; R. Batten leg.; NBCL, RMNH.INS.1485991 • 1 female; Fthiotis, 20 km S. of Lamia; 4 Jul. 1977; R. Batten leg.; NBCL, RMNH.INS.1485992 • 1 female; Aitolia, 5 km W of Navpaktos; 8 Jul. 1977; R. Batten leg.; NBCL, RMNH.INS.1485993 • 1 male; Aitolia, 11 km S of Agrinion; 8 Jul. 1977; R. Batten leg.; NBCL, RMNH.INS.1485998 • 1 female; Arta, 12 km NW of Arta; 8 Jul. 1977; R. Batten leg.; NBCL, RMNH.INS.1485994 • 1 male; Lakonia, 10 km N Sparti; 7 Jul. 1978; R. Batten leg.; NBCL, RMNH.INS.1485995 • 1 male, 1 female; Attica; E. Reitter leg.; syntypes of *Mordellistenagemellata* Schilsky, 1898; MNB • 1 female; “Parnass”; syntype of *Mordellistenagemellata* Schilsky, 1898; D. Krüper leg.; MNB.

#### Additional material examined.

Cyprus • 2 males, 3 females; Choirokoitia; 34.795833°N, 33.337500°E; alt. 186 m; 15 Jul. 2009; M. Mantič leg.; forest steppe, on flowers; MMCZ • 2 males; Pegeia, Coral Bay; 34.858333°N, 32.364167°E; alt. 9 m; 21 May 2017; M. Mantič leg.; forest steppe by sea, on flowers; MMCZ • 1 male, 6 females; Foinikaria, Germasogeia Reservoir; 34.755278°N, 33.093333°E; alt. 68 m; 27 Apr. 2018; D. Selnekovič leg.; secondary grassland, on *Daucus*; DSBS, DSBS15, DSBS16 • 1 male, 1 female; Kouklia env.; 34.666474°N, 32.628931°E; alt. 34 m; 9 May 2022; D. Selnekovič leg.; xeric grasslands on slopes, on *Ferula*; DSBS, DSBS609, DSBS610 • 1 male; Potamiou env.; 34.818289°N, 32.797439°E; alt. 677 m; 10 May 2022; D. Selnekovič leg.; road verge, orchards, on *Daucus* and *Tordylium*; DSBS, DSBS611 • 1 female; Pissouri; 34.653149°N, 32.715005°E; alt. 29 m; 12 May 2022; D. Selnekovič leg.; crop fields, slopes with xeric vegetation, on *Ferula* and *Tordylium*; DSBS • 3 males, 4 females; Avdimou env.; 34.675610°N, 32.758667°E; alt. 33 m; 13 May 2022; D. Selnekovič leg.; road verge, crop fields, on *Daucus* and *Ferula*; DSBS • 2 females; Kyrenia, Bellapais; 13 Jul 1939; H. Lindberg leg.; MZH, http://id.luomus.fi/GAC.38997. Greece • 6 specimens; Crete, Paralia Kouma; 35.352222°N, 24.298889°E; alt. 1 m; 16 Jul. 2012; M. Mantič leg.; sand beech, on flowers; MMCZ • 7 specimens; Crete, Paralia Preveli; 35.152778°N, 24.473333°E; alt. 7 m; 5 Jun. 2018; M. Mantič leg.; forest steppe, on flowers; MMCZ • 4 specimens; Crete, Lavris, 4 km W of Panormos; 35.417778°N, 24.648611°E; alt. 21 m; 1 Jun. 2018; M. Mantič leg.; forest steppe, on flowers; MMCZ • 1 female; Crete, Panormos env.; 35.416667°N, 24.689444°E; alt. 27 m; 30 May 2018; M. Mantič leg.; forest steppe, on flowers; MMCZ • 1 female; Crete, Orino Gorge, 1.8 km NW of Koutsoras; 35.045278°N, 25.932778°E; alt. 97 m; 19 May 2023; M. Mantič leg.; stram valley, on flowers; MMCZ • 4 specimens; Crete, Ierapetra; 35.014167°N, 25.768333°E; alt. 20 m; 16 May 2023; M. Mantič leg.; olive groves, on flowers; MMCZ • 1 male; Crete, Chochlakies; 35.146389°N, 26.246389°E; alt. 106 m; 10 Jun. 2023; M. Mantič leg.; olive groves, on flowers; MMCZ • 1 male; Crete, Kournas, Kourna lake; 30 Jun. 2002; A. Přidal leg.; NMPC • 1 male; Crete, Knossos; 1934; Mařan and Štěpánek leg.; NMPC • 2 specimens; Rhodes, Kalythiés env.; 36.326339°N, 28.187810°E; alt. 43 m; 26 May 2023; D. Selnekovič leg.; olive orchard, on *Ammimajus* and *Helichrysum* sp.; DSBS, DSBS715B, DSBS716 • 4 males, 1 female; Rhodes, Afantou env.; 36.311961°N, 28.189384°E; alt. 9 m; 26–30 May 2023; D. Selnekovič leg.; ruderal community, on *Daucus* sp.; DSBS • 3 males, 3 females; Rhodes, Kolympia; 36.259536°N, 28.131744°E; alt. 49 m; 28 May 2023; D. Selnekovič leg.; olive orchard, on *Daucus* and *Ammimajus*; DSBS, DSBS623 to DSBS626 • 11 specimens; Rhodes, Kolympia; 36.252222°N, 28.168056°E; alt. 15 m; 21 May 2011; M. Mantič leg.; forest steppe, on Apiaceae; MMCZ • 2 females; Rhodes, Kalythiés env.; 36.3202469°N, 28.1867823°E; alt. 14 m; 1 Jun. 2023; D. Selnekovič leg.; ruderal grassland with olive trees and shrubs, on *Daucus* sp.; DSBS. Italy • 4 males, 1 female; Campania, Naples, Camaldoli, 40.852285°N, 14.202138°E; alt. 142 m; 3 Jul. 2023; D. Selnekovič leg.; ruderal habitat, on *Daucuscarota*; DSBS, DSBS558, DSBS598, DSBS599 • 5 males; Sardinia, Sassari; 40.712163°N, 8.549854°E; alt. 207 m; 27 Jun. 2021; D. Selnekovič leg.; urban area, ruderal community along olive orchard and parking lot, on *Daucuscarota*; DSBS, DSBS700 to DSBS704 • 3 males; Puglia, Foggia, Vieste; 41.902976°N, 16.086091°E; alt. 60 m; 3 Jun. 2021; E. Ruzzier leg.; road verge, on *Daucuscarota*; ERPC • 1 male; Sicilia, Caltagirone; NMPC. Turkey • 1 male, 1 female; Mugla, Bodrum, Aspat; 24 May 2008; N. Gulpercin leg.; LEMT • 1 female; Mugla, Bodrum, Aspat; 7 Jun. 2008; S. Tezcan leg.; LEMT.

#### Differential diagnosis.

**Figure 1.**
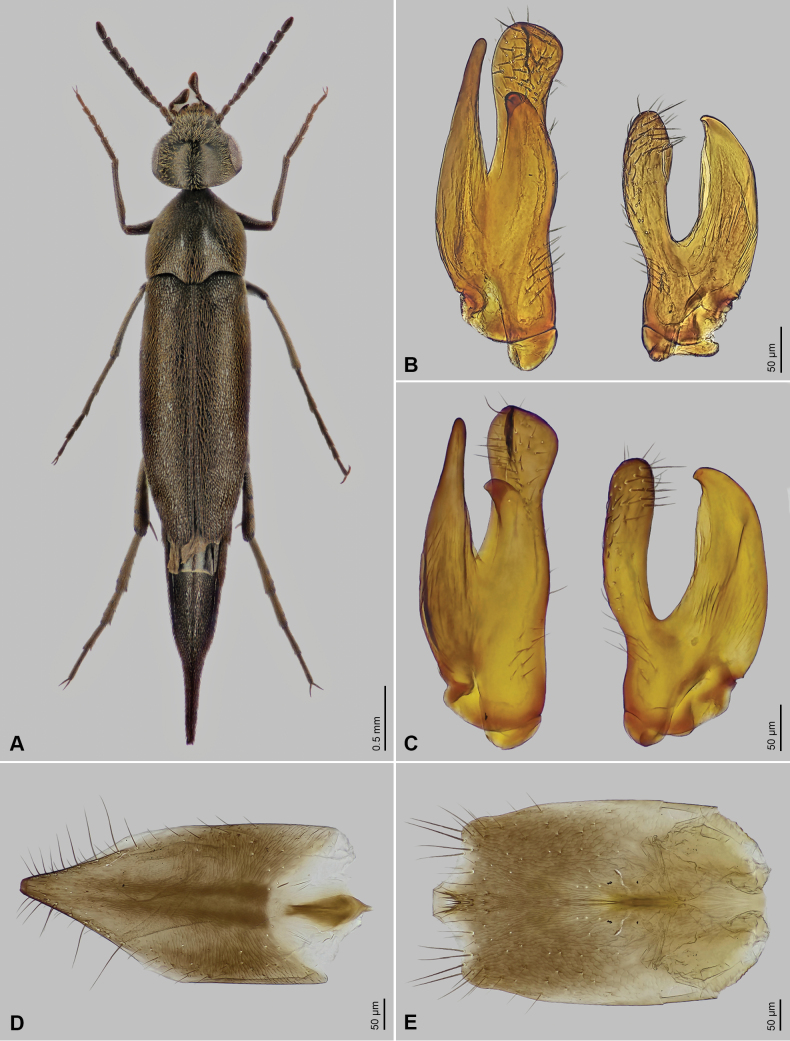
*Mordellistenapeloponnesensis* Batten, 1980 **A** habitus, male, Rhodes, Greece, DSBS623 **B** parameres, paratype from Skala (type locality), RMNH.INS.1485999 **C** parameres, specimen from Italy, DSBS558 **D** female sternite VIII, DSBS610 **E** male sternite VIII, DSBS558.

*Mordellistenapeloponnesensis* is characterised by antennomeres 1–4 being narrower than the following ones and by the presence of two short lateral ctenidia on the posterior tibia that are perpendicular to the dorsal edge of the tibia. This combination of characters is unique to the *M.gemellata* species group as defined by Ermisch (1956). Within this group, *M.peloponnesensis* (Fig. 1) is further distinguished by its black integument, including mouth parts and appendages, pale brownish to yellowish pubescence on the dorsal surface of the body, short antennal segments 5–10 (1.2–1.5× longer than wide), relatively large body size (TL 3.26–4.87 mm), long and narrow elytra (2.2–2.5× longer than wide), male tibia expanded at the base with a group of extended setae, and the characteristic shape of parameres (Fig. 1B, C).

The species most closely resembling *M.peloponnesensis* in terms of size, body shape, elytral length and width, and pubescence color are *M.algeriensis* Ermisch, 1966 (Algeria, Italy, and Tunisia), *M.gemellata* Schilsky, 1898 (Portugal, Spain), and *M.pyrenaea* Ermisch, 1966 (France, Spain). These species can be differentiated from *M.peloponnesensis* by distinct paramere shapes [see Selnekovič and Kodada (2022) for *M.algeriensis*; Ermisch (1966) for *M.pyrenaea*; and Ermisch (1963a) for *M.gemellata*]. Of these, *M.algeriensis*, recently reported from Sardinia (Selnekovič and Kodada 2022), is the only species with a range that partially overlaps with *M.peloponnesensis*. It differs from *M.peloponnesensis* not only in paramere shape but also in size: *M.algeriensis* has an EL/RPrL ratio of 5.89–6.42 and an EL/LPrL ratio of 5.21–5.54, while *M.peloponnesensis* has corresponding ratios of 6.84–8.61 and 5.65–7.38. Additionally, *M.algeriensis* can be distinguished by its subquadrate antennal segments 5–10 (~1.0× longer than wide), whereas in *M.peloponnesensis*, these segments are 1.2–1.5× longer than wide. *Mordellistenaaureotomentosa* Ermisch, 1966, described from Morocco, differs from *M.peloponnesensis* by its conspicuously light-yellowish and dense pubescence on the pronotum and elytra, and smaller, differently shaped parameres (Ermisch 1966). *Mordellistenamaroccana* Ermisch, 1966, found in Morocco and Tunisia, differs from *M.peloponnesensis* in the distinctly shorter and broader elytra: the elytral length/width ratio in *M.maroccana* is 2.0, while in *M.peloponnesensis* it is 2.2–2.5.

#### Redescription.

Measurements (in mm; ♂♂ *N* = 13, ♀♀ *N* = 10): TL: ♂♂ 3.26–4.52 (3.91 ± 0.45), ♀♀ 3.98–4.87 (4.41 ± 0.32); HL: ♂♂ 0.61–0.80 (0.72 ± 0.07), ♀♀ 0.72–0.85 (0.79 ± 0.05); HW: ♂♂ 0.65–0.84 (0.76 ± 0.07), ♀♀ 0.72–0.94 (0.84 ± 0.07); PL: ♂♂ 0.75–1.06 (0.91 ± 0.12), ♀♀ 0.88–1.19 (1.04 ± 0.10); PW: ♂♂ 0.73–1.06 (0.91 ± 0.12), ♀♀ 0.86–1.17 (1.05 ± 0.09); EL: ♂♂ 1.91–2.68 (2.28 ± 0.27), ♀♀ 2.35–2.82 (2.58 ± 0.17); EW: ♂♂ 0.77–1.14 (0.95 ± 0.12), ♀♀ 0.97–1.25 (1.12 ± 0.09); RPrL: 0.25–0.32 (0.30–0.02); LPrL: 0.31–0.41 (0.36 ± 0.03).

Body elongate (Fig. 1A), wedge-shaped, widest behind anterior third of elytra in males, around elytral mid-length in females. Dorsum moderately convex, venter strongly so. Entire body surface uniformly black, except for reddish-brown anteclypeus and tips of mandibles. Vestiture consisting of decumbent lanceolate setae; yellowish to brownish with purple sheen on dorsal surfaces, darkened towards elytral apices; yellowish on ventral surfaces, darkened along posterior margins of ventrites 3–5.

Head approximately as long as wide, HW/HL ratio: ♂♂ 1.02–1.12 (1.07 ± 0.03), ♀♀ 0.95–1.11 (1.07 ± 0.04), moderately convex dorsally, with highest point behind middle of eye length (lateral aspect); occipital carina rounded; integument weakly microreticulate, with small round setiferous punctures. Eyes broadly oval, approx. 1.4× longer than wide; posteriorly reaching to occipital margin; finely faceted; interfacetal setae longer than facet diameter. Anterior clypeal edge weakly convex. Labrum transverse, densely setose, anterior margin weakly convex. Antenna weakly serrate (Fig. 1A); antennomeres 1–4 shorter and narrower than following antennomeres; scape weakly conical, little longer than wide; pedicel cylindrical, approx. 1.5× longer than wide; antennomere 3 smallest, conical, as long as wide; antennomere 4 conical, approx. 1.7× as long as 3; antennomeres 5–10 in male 1.4–1.5×, in female 1.2–1.4× longer than wide, antennomere 5 longest; antennomere 11 oval, approx. 1.8× longer than wide; all antennomeres covered with decumbent trichoid sensilla, additionally, antennomeres 5–10 each with several long and erect trichoid sensilla apico-mesially. Galea moderately long, with spatulate sensilla and trichoid sensilla apically. Maxillary palpomere 2 subcylindrical, weakly expanded distally, similarly formed in both sexes except for distinctly longer trichoid sensilla on ventral surface in male; terminal maxillary palpomere scalene triangular, little wider in male than in female, mesial angle just behind mid-length; numerous decumbent and several erect trichoid sensilla over entire surface; apical sensory area with numerous short sensilla.

Pronotum approximately as long as wide, PL/PW ratio: ♂♂ 0.93–1.09 (1.00 ± 0.04), ♀♀ 0.96–1.02 (1.00 ± 0.02), widest before middle, strongly convex; surface microreticulate, densely covered with lanceolate setae, punctures larger than those on head; anterior edge convex in middle, anterior angles broadly rounded; lateral carinae strongly sinuate in lateral aspect; posterior edge sinuate, posterior angles rectangular in lateral aspect. Scutellar shield triangular, densely setose. Elytra moderately long and narrow, EL/EW ratio: ♂♂ 2.23–2.52 (2.40 ± 0.08), ♀♀ 2.18–2.42 (2.30 ± 0.07); apices separately rounded; surface microreticulate, densely covered with decumbent lanceolate setae, punctures coarser than those on pronotum. Hindwing fully developed. Metanepisternum approx. 4× longer than maximum width, narrowed posteriorly. Protarsomeres cylindrical, each little narrower than preceding one; protarsomere 1 as long as two following tarsomeres combined; penultimate protarsomere truncate distally, with apical edge weakly concave; each protarsal claw with three denticles; male protarsus with longer and thicker seta on ventral surface than female one. Mesotibia approx. 0.8× as long as mesotarsus. Mesotarsomeres cylindrical, each little narrower than preceding one; first mesotarsomere as long as two following tarsomeres combined. Metatibia with short subapical ctenidium and two lateral ctenidia nearly perpendicular to dorsal tibial edge, proximal ctenidium located around mid-length of tibia, distal one at around third quarter; outer terminal spur ca. 0.5× as long as inner one. Metatarsomere 1 with three ctenidia; metatarsomere 2 with two ctenidia; metatarsomeres 3 and 4 without lateral ctenidia.

Abdominal ventrite 5 with narrowly rounded apical edge. Pygidium long, conical, bent ventrad, narrowly truncate at apex, approx. 0.5× as long as elytra. Male sternite VIII with sinuate posterior edge, postero-lateral angles and medial portion moderately produced, with long trichoid sensilla (Fig. 1E); female sternite VIII produced at middle of posterior edge, with long trichoid sensilla around lateral edges (Fig. 1D), anterior median strut short, clavate. Phallobase forming sheath around penis; tubular part short; anterior struts approx. 5× as long as tubular part; dorsal apodeme strongly sclerotised. Parameres as in Fig. 1B, C: left paramere longer than right one, EL/LPrL ratio: 5.65–7.38 (6.33 ± 0.58), dorsal process dilated and obliquely subtruncate apically, with numerous trichoid sensilla, ventral process slightly shorter than dorsal one, tapering towards apex, median process short and wide, with cluster of sensilla campaniformia located along its dorsal edge; left paramere with dorsal process rather narrow, rounded apically, with trichoid and campaniform sensilla, ventral process slightly shorter than dorsal one, wide, bent dorsad, EL/RPrL ratio: 6.84–8.61 (7.67 ± 0.65). Penis long, narrow, weakly expanded before apex.

#### Secondary sexual dimorphism.

Females on average slightly larger than males. Males somewhat slenderer than females. Second maxillary palpomere with longer setae in males than in females. Terminal maxillary palpomere slightly narrower in females. Male protibia bears several elongate setae in proximal half, female protibia uniformly setose. Male protarsomeres with numerous thick setae ventrally.

#### DNA sequences.

Eighteen sequences of the COI gene barcoding region were generated and submitted to BOLD (www.boldsystems.org) and GenBank (www.ncbi.nlm.nih.gov/genbank/). Details on voucher specimens as well as accession numbers are listed in the Suppl. material 2.

#### Distribution.

**Figure 2.**
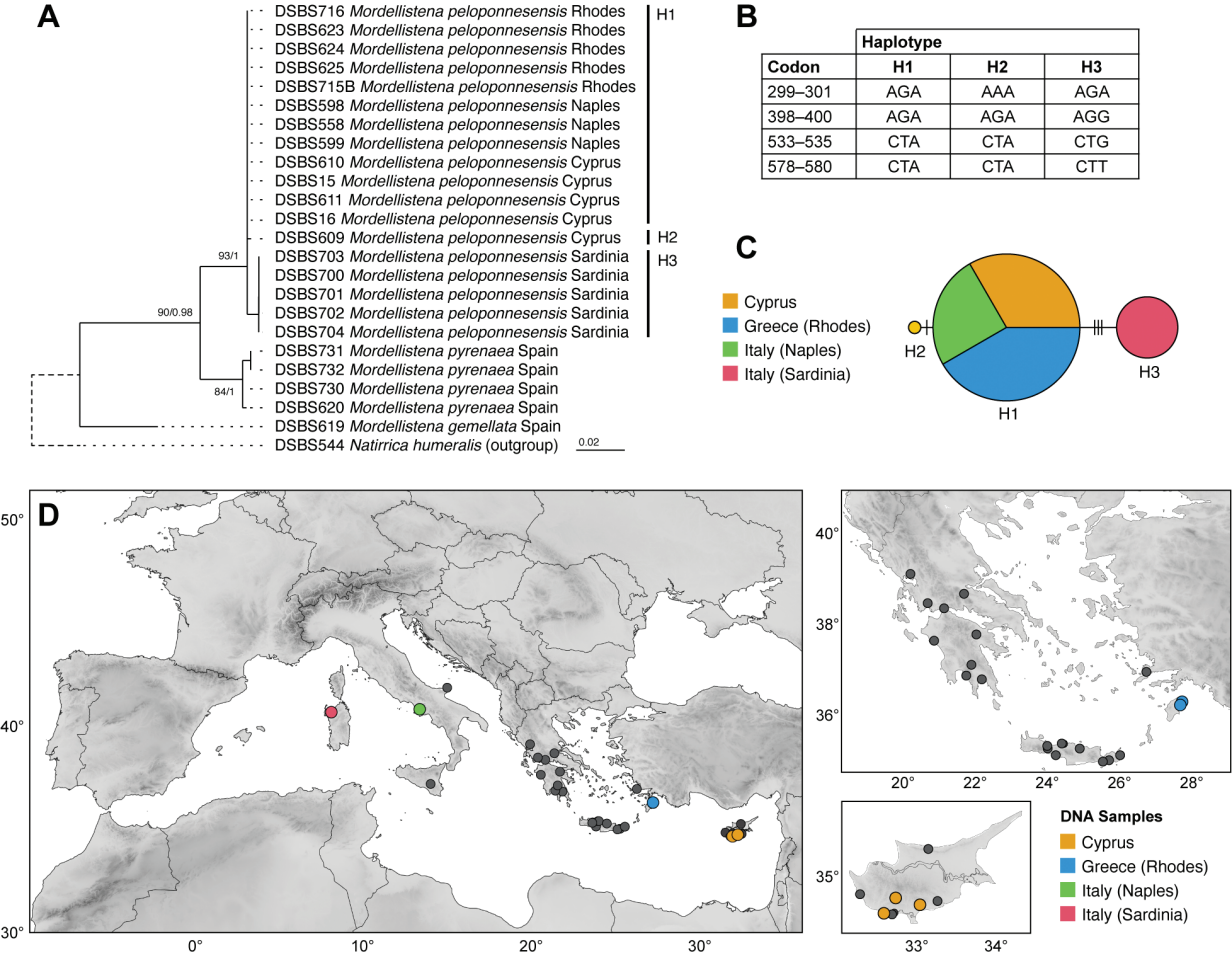
**A** results of maximum likelihood analysis of 658 bp COI fragment; support values are given as: bootstrap values / aBayes test values **B** differences between haplotypes of *Mordellistenapeloponnesensis* Batten, 1980. Codons are marked as the nucleotide positions within 658 bp COI fragment **C** TCS haplotype network based on eighteen sequences of 658 bp COI fragment in *M.peloponnesensis*; colours represent sampling localities; vertical lines represent number of substitutions **D** distribution of *M.peloponnesensis*; left: entire range, right: details on Greece, Turkey, and Cyprus. Localities of DNA samples are marked with coloured dots matching those in the haplotype network; localities without DNA samples are marked with black dots.

Cyprus, Greece (mainland, Crete, Rhodes), Italy (southern part of the Italian peninsula, Sardinia, Sicily), Turkey (Fig. 2D).

#### Habitat and co-occurring species.

Five individuals of *M.peloponnesensis* from Sardinia were sampled in June 2021 from the inflorescences of *Daucuscarota* L. (Apiaceae) in ruderal vegetation separating a parking lot from a small olive orchard in the urban area of Sassari. Three individuals from Vieste were collected on the inflorescences of *D.carota* in ruderal vegetation along a road. Five individuals from the vicinity of Naples were sampled in July 2023 on the inflorescences of *D.carota* in a ruderal habitat along a footpath on Camaldoli Hill. This area featured young secondary forest, orchards, and dry grassland communities. The following species co-occur with *M.peloponnesensis* in Italy: *M.episternalis* Mulsant, 1856, *M.minima* A. Costa, 1854, *M.pseudorhenana* Ermisch, 1977, *M.purpurascens* A. Costa, 1854, *M.tarsata* Mulsant, 1856, *Mediimordabipunctata* (Germar, 1827), *Variimordabasalis* (A. Costa, 1854), and *Stenalia* sp. In similar ruderal habitats along roadsides, secondary grasslands, and olive orchards, specimens of *M.peloponnesensis* were also collected on the islands of Rhodes (Greece) and Cyprus.

##### ﻿DNA analyses

We generated 18 sequences of the COI barcoding region of *M.peloponnesensis* from individuals originating from Cyprus, Greece (Rhodes) and Italy (mainland Italy and Sardinia). We recognised three haplotypes within the species: H1 shared by 12 individuals from Cyprus, Italy (Naples), and Greece (Rhodes); H2 represented by a single individual from Cyprus; and H3 shared by five individuals from Italy (Sardi­nia) (Fig. 2C). H2 differs from H1 at one position in the COI barcoding region (H1—codon 299–301: AGA; H2—codon 299–301: AAA), and H3 differs from H1 at three positions (H1—codon 398–400: AGA, codon 533–535: CTA, codon 578–580: CTA; H3—codon 398–400: AGG, codon 533–535: CTG, codon 578–580: CTT) (Fig. 2B).

We compared the sequences of *M.peloponnesensis* with those of the morphologically allied *M.pyrenaea* (four individuals from Spain) and *M.gemellata* (one individual from Spain). The divergence between *M.peloponnesensis* and *M.gemellata* is 7.94–8.09%, and between *M.peloponnesensis* and *M.pyrenaea* it is 3.34–4.00%. The intra-species divergence within *M.peloponnesensis* reaches a maximum value of 0.61%. Maximum likelihood analysis revealed a separate clade for each species (Fig. 2A).

## ﻿Discussion

*Mordellistenapeloponnesensis* was described by Batten in 1980 based on specimens from various localities in southern mainland Greece and Peloponnese. Since its description, no further specific data have been published, except for its occurrence in Cyprus, as noted in the Catalogue of Palaearctic Coleoptera (Horák 2008, 2020). New data from the islands of Rhodes and Crete, Cyprus, as well as the previously unrecorded distribution of the species in Italy (southern part of the Italian peninsula, Sardinia, Sicily) and Turkey, significantly contribute to understanding this species’ distribution. The records from Italy represent the northernmost and westernmost known occurrences of the species.

DNA sequences of *M.peloponnesensis* are currently available from the vicinity of Naples and Sardinia in Italy, from the island of Rhodes in Greece, and Cyprus. Analysis has shown that individuals from mainland Italy share the same haplotype as individuals from Rhodes and Cyprus. In contrast, individuals from Sardinia possess a distinct haplotype, separated by three nucleotide substitutions. Phylogenetic analysis suggested that the most closely related species is *M.pyrenaea*, known from Spain and France, with an interspecific divergence of 3.34–4.00%. The analysis did not include other morphologically similar species (*M.algeriensis*, *M.aureotomentosa*, *M.maroccana*, *M.elbrusicola* Ermisch, 1969, *M.microgemellata* Ermisch, 1965) for which DNA sequences were not available.

A parallel outcome from the examination of the type material is the identification of two syntypes of *M.gemellata* from Greece (Attica and Parnassus) as *M.peloponnesensis*. Likewise, two specimens from Kyrenia, Cyprus, originally identified and reported by Ermisch (1963b) as *M.gemellata*, are now confirmed to belong to *M.peloponnesensis*. This finding indicates that there are no verified specimens of *M.gemellata* from either Greece or Cyprus, and that the species is reliably documented only from Spain and Portugal (Schilsky 1898; Horák 2008). Additionally, we were unable to confirm a reported record of *M.gemellata* from Estonia (Silfverberg 2004), however, given the species’ known distribution, this record is likely erroneous.

## Supplementary Material

XML Treatment for Mordellistena (s. str.) peloponnesensis

## ﻿Additional information

### Author ORCIDs

Dávid Selnekovič https://orcid.org/0000-0002-9228-1174

Ján Kodada https://orcid.org/0000-0002-1355-4323

Nilay Gülperçin https://orcid.org/0000-0002-9309-6528

Serdar Tezcan https://orcid.org/0000-0003-1980-9291

Enrico Ruzzier https://orcid.org/0000-0003-1020-1247

### Data availability

All of the data that support the findings of this study are available in the main text or Supplementary Information.

## Supplementary materials

Supplementary material 1 Aligned sequences of COI barcoding region Data type fas Explanation note Aligned sequences of 658 bp region of COI used in the present study. https://binary.pensoft.net/file/1146361This dataset is made available under the Open Database License (http://opendatacommons.org/licenses/odbl/1.0/). The Open Database License (ODbL) is a license agreement intended to allow users to freely share, modify, and use this Dataset while maintaining this same freedom for others, provided that the original source and author(s) are credited.Dávid Selnekovič, Ján Kodada, Nilay Gülperçin, Serdar Tezcan, Enrico Ruzzier

Supplementary material 2 List of DNA voucher specimens and sequence accession numbers Data type xlsx Explanation note Linked table containing data on DNA vouchers used in the present study. https://binary.pensoft.net/file/1146362This dataset is made available under the Open Database License (http://opendatacommons.org/licenses/odbl/1.0/). The Open Database License (ODbL) is a license agreement intended to allow users to freely share, modify, and use this Dataset while maintaining this same freedom for others, provided that the original source and author(s) are credited.Dávid Selnekovič, Ján Kodada, Nilay Gülperçin, Serdar Tezcan, Enrico Ruzzier
